# Editorial for Special Issue: Machine Health Monitoring and Fault Diagnosis Techniques

**DOI:** 10.3390/s23073493

**Published:** 2023-03-27

**Authors:** Shilong Sun, Changqing Shen, Dong Wang

**Affiliations:** 1 School of Mechanical Engineering and Automation, Harbin Institute of Technology, Shenzhen 518055, China; 2 Guangdong Provincial Key Laboratory of Intelligent Morphing Mechanisms and Adaptive Robotics, Shenzhen 518055, China; 3 School of Rail Transportation, Soochow University, Suzhou 215131, China; 4 The State Key Laboratory of Mechanical System and Vibration, Shanghai Jiao Tong University, Shanghai 200240, China

Machine health monitoring and fault diagnosis have played crucial roles in automatic and intelligent industrial plants. Machine-learning-, deep-learning-, and artificial-intelligence-based intelligent fault diagnoses are essential in industrial settings, in order to help reduce the downtime that is caused by machine failures. These techniques can be integrated with advanced sensor technologies to enhance the accuracy of their results. Additionally, some specific artificial intelligence algorithms can help to identify potential problems and alert engineers on time. However, there are still several issues that require further investigation, with intelligent fault diagnosis methodologies being among them, e.g., early fault detection features, the few-shot sample machine learning algorithm, data augmentation techniques for deep learning, the data fusion method for domain adaptation, feature representation with self-supervision, and interpretable deep learning algorithms. Ultimately, machine health monitoring and fault diagnosis techniques are essential tools for ensuring a machine’s safety and efficiency.

This Special Issue aims to highlight the state-of-the-art techniques that are used for machine health monitoring and fault diagnosis, especially for intelligent fault diagnosis algorithm development, fault feature extraction, and intelligent machine monitoring.

This Special Issue has received 26 manuscripts, 18 of which have been accepted, and 8 of which were rejected by the peer-review processes. These accepted manuscripts can be divided into four types: (1) status detection; (2) degradation process; (3) fault diagnosis; and (4) failure detection with sensors. Their details have been illustrated as follows:

## 1. Status Detection

In [1], a novel automated algorithm for the modal parameter identification of rotating machinery was described. The innovation of this study was that it targeted a rotor mode and is applicable to different systems and environments. This algorithm extracted the rotor and fundamental frequency damping ratios from a stability diagram that was given a user-defined parameter.

In [2], a multimodal process monitoring method, which was based on variable-length sliding window-mean augmented Dickey–Fuller (VLSW-MADF) test and a dynamic locality-preserving principal component analysis (DLPPCA), was proposed. 

The work that was developed in [3] presented a new algorithm for impeller blade monitoring, based on a relative shaft vibration signal measurement and analysis, which was designed to run from a long-term perspective as part of a remote monitoring system in order to automatically track a natural blade frequency and its amplitude.

In [4], a method using multidimensional k-means for the condition monitoring of electrode wear was established. With the aid of this method, the relationship between the serial time data of the resistance and the mechanical properties variation of the electrodes was described. 

## 2. Degradation Process

A method for evaluating bearing performance degradation, by using an adaptive sensitive feature selection and multi-strategy optimization support vector data description (SVDD), was developed in [5]. In combining the technique for order preference by similarity with an ideal solution (TOPSIS) and K-medoids, monotonicity, correlation, and robustness indicators were used to determine an adaptive sensitive feature set for evaluation. 

In [6], a new cluster migration distance (CMD) algorithm was proposed to address the problem in which traditional performance degradation indicators cannot accurately describe degradation trending on time. By calculating the offset trajectory of a feature cluster centroid in a continuous bearing running process, the CMD can appropriately handle the complex and variable features in the fault evolution process of a water pump bearing. 

In [7], they used a ring oscillator (RO)-based test structure to extract data and build a dataset that could be used to predict aging trends and determine the primary aging mechanisms of 28 nm FPGAs. Moreover, they proposed a method to correct the temperature-induced measurement errors that are found in accelerated tests. Furthermore, they employed four machine learning (ML) technologies that were based on accurate measurement datasets, in order to predict these FPGA aging trends. 

In [8], a method based on an improved particle swarm optimization (PSO) was proposed to analyze the bearing performance degradation. This proposed method can effectively resolve the problems of online parameter selection and the low predictive accuracy of long–short time memory (LSTM) methods. A kernel joint approximate diagonalization of eigen-matrices (KJADE) method was used to fuse the bearing vibration signals and form an effective feature vector, and an SS was calculated to acquire a performance degradation index. Subsequently, an improved PSO algorithm was used to optimize the LSTM parameters, in order to obtain an optimal performance degradation prediction model.

## 3. Fault Diagnosis

In [9], a model for data augmentation was proposed. This study proposed a method for the unbalanced fault diagnosis of rotating machinery that combined time–frequency feature oversampling (TFFO) with a convolutional neural network (CNN). The proposed model built a balanced dataset by simultaneously expanding time domain signals and time–frequency domain features, and by performing a comprehensive data expansion from different dimensions.

The work in [10] proposed a rolling bearing fault diagnosis method that was based on the whale gray wolf optimization algorithm–variational mode decomposition–support vector machine (WGWOA-VMD-SVM), which was designed to solve the unclear fault characteristics of rolling bearing signals, owing to its nonlinear and nonstationary characteristics. A rolling bearing signal was decomposed using variational mode decomposition (VMD), and a support vector machine (SVM) was used as the fault diagnosis model.

In [11], a novel model was proposed for an intelligent bearing fault diagnosis in rotating machinery. The main contribution of this model is the construction of an effective image dataset using a combination of an improved fast kurtogram (IFK) that was based on nonlinear mode decomposition (NMD) and a gramian angular field (GAF). The proposed model used IFK to achieve a high computational efficiency and improve its SNR. Next, GAF provided images that preserved the absolute temporal relationships of the signals, so that the CNN could perform a fault classification. 

In [12], a novel intelligent rolling bearing fault diagnosis method, based on a Markov transition field (MTF) and a residual network, was proposed. Encoding one-dimensional time series signals into two-dimensional images with a Markov transition field preserved the time dependence of the raw signals and discarded the prior knowledge, in order to set the parameters during the conversion. On this basis, a residual network was applied to identify the fault types through image classification.

In [13], a rolling bearing fault diagnosis method was proposed based on successive variational mode decomposition (SVMD) and an energy concentration and position accuracy (EP) index. The EP index effectively indicated a target mode for the characteristic fault information, and a line-searching method that was guided by the EP index optimized the balancing parameter of the SVMD.

In [14], a method of compressing and reconstructing diesel engine vibration signals was proposed by using sparse Bayesian optimization block learning, which combined a compressive sensing technology with the fault diagnosis. Its specific steps were as follows. The method achieved the optimal compression and reconstruction efficiency, was verified by several assessment indicators, and had a good classification accuracy. However, there was still room for improvement, particularly in the signal repair and noise reduction preprocessing, as well as in the integration of the algorithm into the data acquisition hardware.

In [15], an integrated vision transformer (ViT) model, which was based on wavelet transform and a soft voting method for the bearing fault diagnosis, was proposed. A vibration signal was decomposed into sub-signals in different frequency bands using discrete wavelet transform (DWT), and was transformed into time–frequency representation (TFR) maps using continuous wavelet transform (CWT). Multiple individual ViT models were used to preliminarily diagnose the faults, and a final diagnosis result was obtained by a fusion method that was based on the soft voting method. 

## 4. Failure Detection with Sensor

In [16], the authors analyzed the heat generation of normal bearings and faulty bearings during the operation, and the influence of different working conditions on the heat generation of these bearings. In this study, based on the structural characteristics of the bearings, a new transient temperature analysis model for damaged bearings was established, considering the influence of the thermal–solid coupling effect on the bearing structure. 

In [17], the authors analyzed different types of sensor faults for the fault detection of a healthy drive, using a variety of index-based methods. In total, seven main indices were employed and analyzed for the sensor fault diagnosis, including the moving mean, average, root mean square, energy, variance, the first-order derivative, the second-order derivative, and an auto-correlation-based index. 

In [18], a novel virtual sensor for predictive maintenance, which was called a mini-term, was introduced. One of its main advantages was that its installation did not involve a large financial outlay. The evolution of the TAV (technical availability), mean time to repair (MTTR), EM (number of work orders (emergency orders/line stop)), and OM (labor hours in EM) showed a very important improvement, as the number of mini-terms increased and the Miniterm 4.0 system became more reliable.

## 5. Conclusions

The theme of this Special Issue focuses on machine health monitoring and fault diagnosis techniques, especially intelligent fault diagnosis. This Special Issue highlights 18 articles that can be divided into four categories: condition monitoring [1,2,3,4], degradation process prediction [5,6,7,8], intelligent diagnostic algorithms [9,10,11,12,13,14,15], and sensor fault detection [16,17,18]. In addition to the traditional bearing vibration signals, the research objects include the electrode signals, blade vibration signals, diesel engine vibration signals, and bearing heat signals. Therefore, in the field of fault diagnosis, in addition to the traditional bearing vibration signal analysis, other objects or signals can also be used as diagnostic features, which is worth studying. Regarding the algorithm design, the development of artificial intelligence algorithms also provides new solutions for other signal analyses and processing. Artificial intelligence algorithms and multi-sensor signals, combined with intelligent fault diagnosis algorithms, will be a very important development trend in the future.
